# Combined Spatial Prediction of Schistosomiasis and Soil-Transmitted Helminthiasis in Sierra Leone: A Tool for Integrated Disease Control

**DOI:** 10.1371/journal.pntd.0001694

**Published:** 2012-06-19

**Authors:** Mary H. Hodges, Ricardo J. Soares Magalhães, Jusufu Paye, Joseph B. Koroma, Mustapha Sonnie, Archie Clements, Yaobi Zhang

**Affiliations:** 1 Helen Keller International, Freetown, Sierra Leone; 2 School of Population Health, University of Queensland, Herston, Queensland, Australia; 3 National Neglected Tropical Disease Control Program, Ministry of Health and Sanitation, Freetown, Sierra Leone; 4 Regional Office for Africa, Helen Keller International, Dakar, Senegal; Centre Suisse de Recherches Scientifiques, Côte d'Ivoire

## Abstract

**Background:**

A national mapping of *Schistosoma haematobium* was conducted in Sierra Leone before the mass drug administration (MDA) with praziquantel. Together with the separate mapping of *S. mansoni* and soil-transmitted helminths, the national control programme was able to plan the MDA strategies according to the World Health Organization guidelines for preventive chemotherapy for these diseases.

**Methodology/Principal Findings:**

A total of 52 sites/schools were selected according to prior knowledge of *S. haematobium* endemicity taking into account a good spatial coverage within each district, and a total of 2293 children aged 9–14 years were examined. Spatial analysis showed that *S. haematobium* is heterogeneously distributed in the country with significant spatial clustering in the central and eastern regions of the country, most prevalent in Bo (24.6% and 8.79 eggs/10 ml), Koinadugu (20.4% and 3.53 eggs/10 ml) and Kono (25.3% and 7.91 eggs/10 ml) districts. By combining this map with the previously reported maps on intestinal schistosomiasis using a simple probabilistic model, the combined schistosomiasis prevalence map highlights the presence of high-risk communities in an extensive area in the northeastern half of the country. By further combining the hookworm prevalence map, the at-risk population of school-age children requiring integrated schistosomiasis/soil-transmitted helminth treatment regimens according to the coendemicity was estimated.

**Conclusions/Significance:**

The first comprehensive national mapping of urogenital schistosomiasis in Sierra Leone was conducted. Using a new method for calculating the combined prevalence of schistosomiasis using estimates from two separate surveys, we provided a robust coendemicity mapping for overall urogenital and intestinal schistosomiasis. We also produced a coendemicity map of schistosomiasis and hookworm. These coendemicity maps can be used to guide the decision making for MDA strategies in combination with the local knowledge and programme needs.

## Author Summary

Two forms of schistosomiasis or bilharzia (intestinal and urogenital) exist in Sierra Leone. The main control strategy for this disease currently is through mass drug administration (MDA) according to the World Health Organization recommended anthelminthic chemotherapy guidelines, and others include snail control, behavior change, and safe water, sanitation and hygiene. Survey on distribution and prevalence of the disease is vital to the planning of MDA in each district. The distribution of intestinal schistosomiasis in the country has been reported previously. The current national survey showed that urogenital schistosomiasis has a specific focal distribution particularly in the central and eastern regions of the country, most prevalent in Bo (24.6%), Koinadugu (20.4%) and Kono (25.3%) districts. Using a simple probabilistic model, this map was combined with the previously reported maps on intestinal schistosomiasis and the combined schistosomiasis prevalence was estimated. The combined schistosomiasis map highlights the presence of high-risk communities in an extensive area in the northeastern half of the country, which provides a tool for planning the national MDA activities.

## Introduction

Schistosomiasis or bilharzia is prevalent in 76 countries and territories in tropical and subtropical regions and is estimated to infect over 200 million people worldwide, causing significant morbidity [1], [2]. The disease is caused by infection with trematodes of the *Schistosoma* genus. There are three major species which infect humans: *Schistosoma haematobium* causing urogenital (formerly known as urinary) schistosomiasis, and *Schistosoma mansoni* and *Schistosoma japonicum* (the latter in Asia) causing intestinal schistosomiasis. The geographical distribution of schistosomiasis is dependent on the presence of suitable intermediate host snails in the aquatic environment in the tropics and subtropics, amongst other factors. Hookworm is one of the major soil-transmitted helminthes (STH), infecting 576 million people worldwide and causing anemia and undernutrition particularly in poor rural settings [3].

Both intestinal and urogenital forms of schistosomiasis and hookworm are known to be endemic in Sierra Leone [4], [5], [6], [7], [8], [9], [10]. In 2008, a national integrated control program against neglected tropical diseases (NTDs) including lymphatic filariasis, onchocerciasis, schistosomiasis and STH was initiated with financial support from the United States Agency for International Development (USAID) NTD Control Program managed by RTI International and technical support from Helen Keller International. The programme uses the integrated mass drug administration (MDA) strategy according to the preventive chemotherapy (PCT) guidelines recommended by the World Health Organization (WHO) [11]. To facilitate the planning and implementation of MDA activities, a national mapping survey on prevalence and distribution of schistosomiasis and STH was conducted in 2008 and 2009 [9], [10]. The results showed that *S. mansoni* and hookworm were widespread in Sierra Leone, with high prevalence of *S. mansoni* in Kono, Koinadugu, Kailahun, Kenema and Tonkolili districts and with high prevalence of hookworm across the country. Spatial analysis predicted that there was a large cluster of high risk of *S. mansoni* infection (prevalence >70%) in the north and most of the eastern areas of the country and a large cluster of high risk of hookworm infection (prevalence >70%) in the north-eastern part of the country [9]. However, in that survey, urogenital schistosomiasis was not properly diagnosed due to the limited human resources.

Urogenital schistosomiasis was first reported from Sierra Leone in 1909 [8], and since then, numerous foci of *S. haematobium* have been reported, with varying levels of prevalence [4], [5], [8], [12], [13], [14], [15]. In general, prevalence in Eastern province was relatively high and in Northern and Southern provinces was relatively low. To enable the national integrated NTD control programme to fine tune the praziquantel distribution strategy in each district, a further national survey of urogenital schistosomiasis was conducted before praziquantel distribution in 2009–2010.

A number of methods are available for mapping the codistribution of helminth infections [16]. One of these methods is the production of coendemicity maps [17]. In this paper, we aimed to analyze the newly collected urogenital schistosomiasis dataset to provide the first urogenital schistosomiasis distribution map for the country. Additionally, by combining this map with the previously reported maps on intestinal schistosomiasis and STH, we aimed to estimate the at-risk population of school-age children requiring integrated schistosomiasis/STH treatment regimens according to the coendemicity of these diseases, based on the WHO guidelines.

## Materials and Methods

### Ethics Statement

The national NTD control programme is managed and implemented by the Ministry of Health and Sanitation, Sierra Leone. The programme undertook a national survey on prevalence of each NTD in order to plan the implementation strategy. Ethical approval for data collection in school children was obtained from the Ethics Committee of the Ministry of Health and Sanitation of Sierra Leone. Upon arrival at the selected schools, the investigating team met with the community teachers association in each school, and explained the nature of the survey. Informed consent was verbally given by guardians/parents and recorded by the team leader. The verbal consent was approved by the Ethics Committee as literacy rates are low in Sierra Leone. Once data were collected, the results were entered into a database and analyzed anonymously. No personal identity can be revealed upon publication. All participants subsequently received treatment from the national programme.

### Sampling

Sierra Leone is divided into 12 rural health districts (each with 7–16 chiefdoms) plus rural Western Area (WA) and urban WA. The survey was carried out in 2009 in six rural districts (Bo, Bombali, Kenema, Koinadugu, Kono and Tonkolili) which qualified for mass praziquantel distribution according to *S. mansoni* mapping [9], [10], and in 2010 in seven coastal districts including rural and urban WAs which did not qualify for mass praziquantel distribution according to *S. mansoni* mapping. Due to the focal nature of *S. haematobium* according to the historical data and the programme planning need in Sierra Leone, the survey sites were not selected randomly, but according to the prior local knowledge and local ecological environment in each district, where the villages were thought most likely to have schistosomiasis. To ensure a good spatial coverage of the survey sites within a district, one site was selected from each chiefdom where *S. haematobium* was suspected to be likely endemic. The number of sites surveyed in each district is shown in Table 1. The survey was conducted in primary schools. Within each school, 30–50 children aged 9–14 years were enrolled, balancing for sex. The sample size for each survey site was chosen according to the recommendations in the best practice paper [18], and the WHO guidelines [19]. A total of 52 sites/schools were surveyed and 2293 children (1234 boys and 1059 girls) were examined.

**Table 1:** *Schistosoma haematobium* prevalence and intensity of infection in schoolchildren by district in Sierra Leone.

District	No of sites surveyed	No of children examined	Prevalence (%)(95% CI, minimum-maximum)	Arithmetic mean intensity of infection (eggs/10 ml) (95% CI)
Bo	13	675	24.6 (21.5–28.0, 6.0–48.0)	8.79 (5.69–11.88)
Bombali	9	261	5.7 (3.5–9.3, 0–40.7)	1.34 (0.0–3.04)
Bonthe	2	106	0	-
Kambia	2	104	1 (0.2–5.3, 0–2)	0.06 (0.0–0.18)
Kenema	2	60	0	-
Koinadugu	5	230	20.4 (15.7–26.1, 0–56.3)	3.53 (0.0–9.70)
Kono	6	253	25.3 (20.3–31.0, 16.7–31.0)	7.91 (2.77–13.04)
Moyamba	2	105	0	-
Port Loko	2	100	2 (0.6–7.0, 2–2)	0.22 (0.02–0.42)
Pujehun	2	103	0	-
Tonkolili	3	89	0	-
Rural WA	2	105	1 (0.2–5.2, 0–2)	0.01 (0.0–0.03)
Urban WA	2	102	0	-

The sampling method for *S. mansoni* and STH mapping sites in 2008 has been described previously [9]. Briefly, the survey sites (schools) were selected according to administrative districts (four schools per district) using a two-staged random sampling method to avoid two schools being selected from the same chiefdom to ensure a relatively even geographical coverage throughout the country. In each district four chiefdoms were first randomly selected. Within each selected chiefdom, one primary school was randomly selected. In total, 53 schools were selected for survey throughout the country. Approximately 100 children aged 5 to 16 years per school (range: 36–134) were examined.

### Data Collection and Analysis

One urine sample was collected from each of 2293 children around midday. Each sample container was labeled with an identification number. For examination, volume of urine samples was measured and urine containers were centrifuged for five minutes [20]. The sediment was transferred onto a glass slide and covered with a cover slip. These were examined under a light microscope, and the number of *S. haematobium* eggs was recorded and intensity of infection expressed as number of eggs per 10 ml of urine (eggs/10 ml). The data collection for *S. mansoni* and STHs using the standard Kato-Katz method has already been described in the previous publication [9].

Survey results were entered into Microsoft Excel. Prevalence of infection and corresponding differences between ages and sex were estimated taking into account the clustered nature of the sampling, using the village/school as a primary sampling unit and including adjustments for the probability of sampling and finite population corrections for sampling without replacement in the Stata/SE 10.0 statistical package (StataCorp, College Station, Texas, USA).

The coordinates of each sample site were recorded using hand-held global positioning system (GPS) devices (available upon request). Prevalence at each location was plotted in a geographical information system (GIS) (ArcGIS version 10.0, ESRI, Redlands, CA). Electronic data for land surface temperature (LST) and normalised difference vegetation index (NDVI) for a 5 km×5 km grid cell resolution were obtained from the National Oceanographic and Atmospheric Administration's (NOAA) Advanced Very High Radiometer (AVHRR; see Hay et al. [21] for details on these datasets) and the location of large perennial inland water bodies (PIWB) was obtained from the Food and Agriculture Organization of the United Nations (http://www.fao.org/geonetwork/srv/en/main.home) and the distance to PIWB was extracted for each survey location in the GIS. A 5 km resolution population surface derived from the Global Rural-Urban Mapping Project (GRUMP) beta product was obtained from the Center for International Earth Science Information Network (CIESIN) of the Earth Institute at Columbia University (http://sedac.ciesin.columbia.edu/gpw/global.jsp). Elevation data with a 5 km×5 km grid resolution, generated by a digital elevation model (DEM) from the Shuttle Radar Topography Mission (SRTM), were obtained from the Global Land Cover Facility (http://glcf.umiacs.umd.edu/index.shtml). All environmental datasets were linked to survey locations and values at each survey location were extracted in the GIS.

### Framework of Analysis

**Figure 1:**
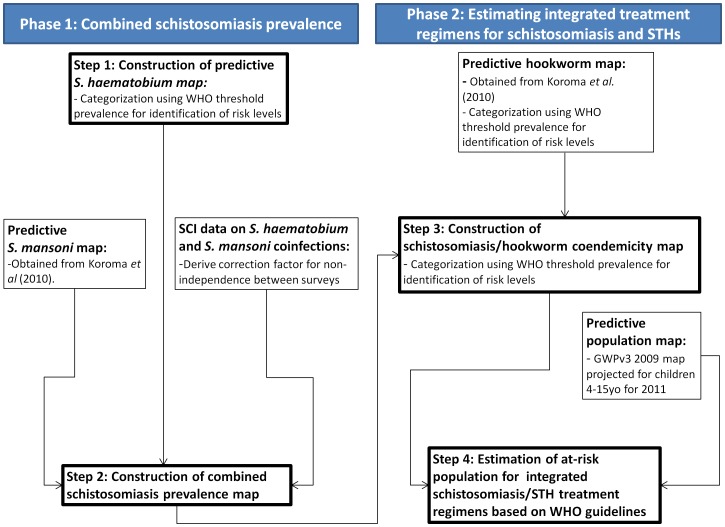
Framework of analysis.

The analysis was carried out in two phases (Figure 1): in the first phase we aimed to quantify the combined schistosomiasis prevalence. We developed a predictive map of *S. haematobium* prevalence for Sierra Leone using model-based geostatistics. The resulting *S. haematobium* predictive map was combined with an existing predictive map of *S. mansoni* prevalence using a probabilistic approach (see below) to obtain a combined urogenital and intestinal schistosomiasis map. This map was then categorized based on the prevalence thresholds in the WHO guidelines for praziquantel distribution: low-risk communities for schistosomiasis were defined as those in areas that had combined prevalence of both infections <10%, moderate-risk communities in areas having combined prevalence of both infections 10–50% and high-risk communities in areas having combined prevalence of both infections >50%. This allowed the identification of areas in Sierra Leone by schistosomiasis risk level.

In the second phase of the analysis we combined risk maps of schistosomiasis and major STH (hookworm) to quantify the population requiring the different WHO recommended treatment regimens for each parasite. We overlaid the combined schistosomiasis map generated in Phase 1 with an existing map of hookworm prevalence to obtain a schistosomiasis/hookworm co-endemicity map. Hookworm was chosen for this analysis because in a recent nationwide parasitological survey, hookworm was the STH with the highest prevalence (38.5%) in the country [9], [10], and the prevalence of the other major STH (*Ascaris lumbricoides*, 6.6% and *Trichuris trichiura*, 1.8%) was too low to warrant risk mapping. The hookworm prevalence map was categorized into the WHO prevalence thresholds that define risk levels for STH infection and appropriate albendazole treatment regimens (20–50% and >50%). The resulting coendemicity map was then overlaid with the GRUMP population map in the GIS and the population size in areas belonging to a given coendemicity class was then calculated in order to obtain the numbers of individuals at each risk level of both infections.

### *S. haematobium* Spatial Risk Prediction

The initial candidate set of predictor variables included population density, LST, NDVI, PIWB and elevation. Fixed-effects binomial logistic regression models of prevalence of *S. haematobium* infection were developed in a frequentist statistical software package (Stata version 10.1, Stata Corporation, College Station, TX). In the preliminary, non-spatial multivariable model, elevation was not found to be significantly associated with *S. haematobium* infection risk (Wald's *P*>0.2) and this variable was excluded from further analysis. A quadratic association between LST and prevalence of infection was assessed and was not found to improve model fit using the Akaike's Information Criterion. Residual spatial dependence was investigated using semivariograms using the package geoR of the statistical software R.

We developed the model-based geostatistical spatial prediction model [22] for *S. haematobium* using the Bayesian statistical software, WinBUGS version 1.4 (Medical Research Council Biostatistics Unit, Cambridge, United Kingdom and Imperial College London, London, United Kingdom). Several models were tested and all had the covariates plus a geostatistical random effect, in which spatial autocorrelation between locations was modeled using an exponentially decaying autocorrelation function. Statistical notation of Bayesian geostatistical models, spatial interpolation and model validation procedures are presented in an additional file (Text S1).

### Estimating Combined Prevalence of Schistosomiasis

We used predicted prevalence estimates of *S. haematobium* and the predicted *S. mansoni* prevalence estimates from Koroma *et al* [9] to derive a combined urogenital and intestinal schistosomiasis prevalence estimate. The combined prevalence was calculated using a simple probabilistic model, incorporating a small correcting factor to allow for non-independence of schistosome species following the approach of de Silva and Hall [23]. In brief, when assuming that the probability of infection with one schistosome species is independent of another, the predicted combined probability of having at least one schistosome infection is the simple probability law for the union between two probabilities:
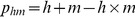
(1)where 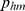 is the combined urinary and intestinal schistosomiasis prevalence, *h* is the urinary schistosomiasis prevalence and *m* is the intestinal schistosomiasis prevalence. This equation was implemented in the GIS and multiplied by a correction factor due to non-independence between both schistosome surveys. Without this correction factor, the predicted combined prevalence of schistosomiasis would be an overestimate. The correction factor was estimated using data from 67 schools with urinary schistosomiasis and intestinal schistosomiasis coinfections in Burkina Faso, Ghana, Mali and Niger, collected between 2007–2008 with support from the Schistosomiasis Control Initiative (SCI) [16], [24], [25]. Using these data we plotted the difference between predicted and observed combined prevalence against the observed combined prevalence in each school. We found the association to be highly non-linear, negating the use of a simple linear equation to describe the correction factor. We then fitted non-linear parametric functions using a function finder interface freely available on the internet (www.zunzun.com). This online resource allows curve fit to non-linear observational and experimental data by comparing and estimating fit statistics to a library of over 500 non-linear functions. The Python code for curve fitting is available on the Google code repository http://code.google.com/p/pythonequations/.

### Estimating the Number of School-Age Children with Schistosomiasis/Hookworm Infections

We used the predicted combined prevalence of schistosomiasis map and the predicted hookworm prevalence estimates from Koroma *et al* [9] to derive a schistosomiasis/hookworm coendemicity map. This map was overlaid in the GIS by a 2011 GRUMP population map for children aged between 5–15 years old in Sierra Leone, projected from a 2009 GRUMP map to obtain the number of school-age children with schistosomiasis/hookworm coinfections. This projection assumed a population growth rate 2005–2011 of 2.60% and proportion of 5–15 years old of 26.5% for 2011 (http://esa.un.org/unpd/wpp/index.htm), that was constant across the country.

## Results

### *S. haematobium* Prevalence Distribution

**Figure 2:**
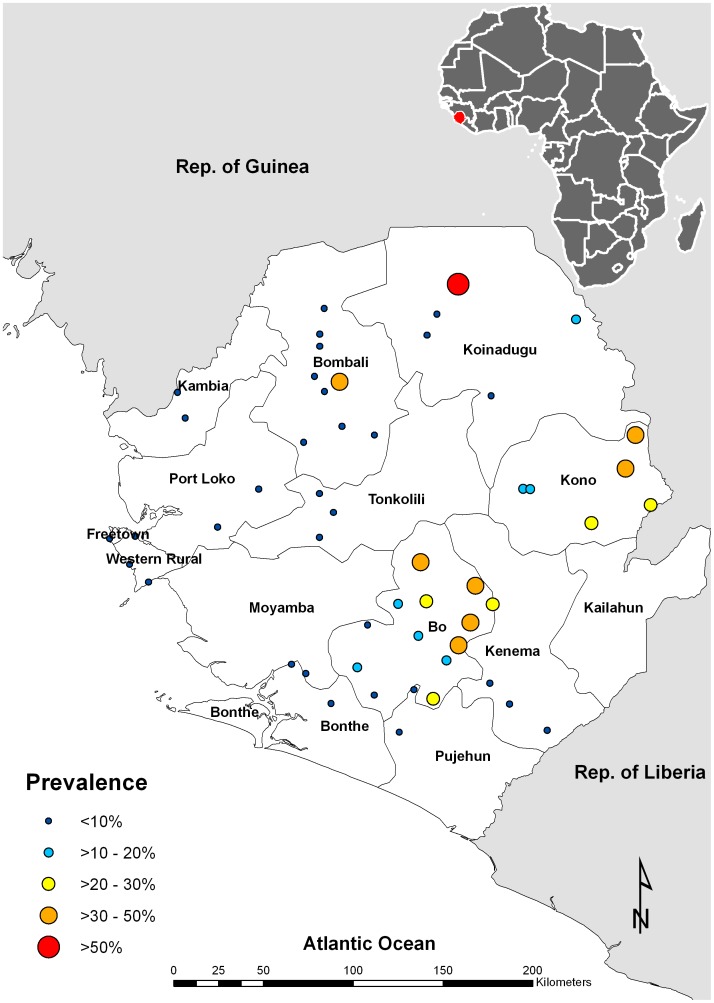
Geographical distribution of urogenital schistosomiasis in Sierra Leone 2010.

Point prevalence of *S. haematobium* infection from each survey site is shown in Figure 2. Across 52 sites surveyed, *S. haematobium* infection was found in 30 sites, mainly in Bo and Kono districts. The median prevalence was 2% (inter-quartile range: 0–18.6% and minimum-maximum range: 0–56.3%) in all sites and 17.7% (inter-quartile range: 6.2–30.5% and minimum-maximum range: 2–56.3%) in *S. haematobium*-positive only sites. Arithmetic mean intensity of infection including all children examined was 3.98 eggs/10 ml urine (95% CI: 2.73–5.22 eggs/10 ml). There was no significant difference in either prevalence or intensity of infection between boys and girls or between ages (p>0.05, details not shown). Table 1 summarizes the prevalence and intensity of infection in each district surveyed. *S. haematobium* was found mainly in the northeast half of the country, with a relatively higher level of endemicity in Bo (24.6% and 8.79 eggs/10 ml), Koinadugu (20.4% and 3.53 eggs/10 ml) and Kono (25.3% and 7.91 eggs/10 ml) districts. Little *S. haematobium* infection was found in the southern and western coastal districts.

### Spatial Risk Prediction of *S. haematobium* Infection

**Figure 3:**
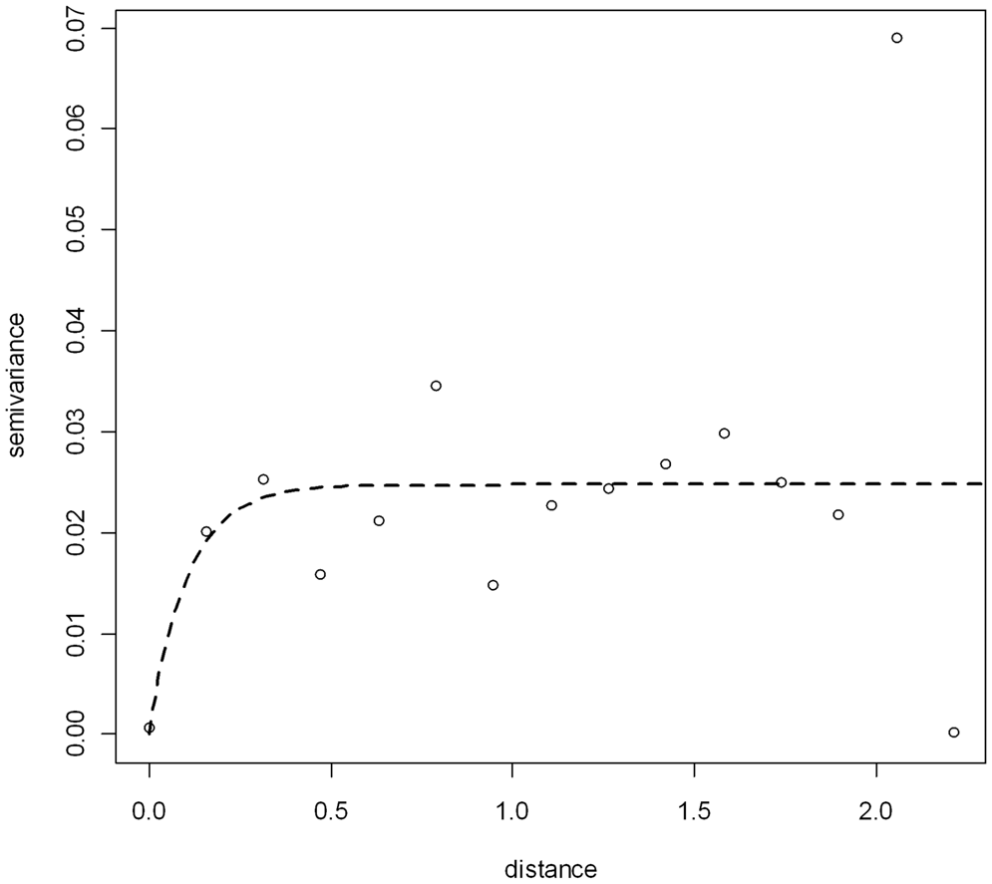
Semivariogram of residual spatial variation in *S. haematobium* prevalence in school-age children, Sierra Leone 2010.

In the non-spatial model of *S. haematobium* infection, the semivariogram of model residuals exhibited strong spatial variation unaccounted for by the variables included in the model (Figure 3), justifying a model-based geostatistical approach. Spatial model results (Table 2) indicated that there was no clear association between prevalence of *S. haematobium* and sex, age, LST, NDVI, PIWB or population density. The rate of decay of spatial autocorrelation [Phi (*φ*)] was 4.20. This indicates that, after accounting for the effect of covariates, the radii of the clusters were approximately 79 km (note, *φ* is measured in decimal degrees and 3/*φ* determines the cluster size; one decimal degree is approximately 111 km at the equator). Variance of the spatial random effect (*σ* ^2^) was 6.41, indicating a strong tendency for spatial clustering.

**Table 2:** Spatial effects for prevalence of *Schistosoma haematobium* infection in schoolchildren in Sierra Leone, 2009–2010.

Variable	Posterior mean (95%CI)
Male (vs female)	−0.08 (−0.38, 0.21)
Age in years	−0.06 (−0.14, 0.03)
LST*	0.59 (−0.96, 2.16)
NDVI*	−0.21 (−1.46, 1.12)
Population density*	0.01 (−1.18, 1.26)
PIWB*	0.53 (−1.26, 3.06)
Intercept	−2.74 (−4.48, −0.99)
*φ* (rate of decay of spatial correlation)	4.20 (0.77, 14.65)
*σ* ^2^ (variance of spatial random effect)	6.41 (2.41, 17.63)

*: Variables were standardised to have mean = 0 and standard deviation = 1; CI = Bayesian credible interval; LST = Land Surface Temperature; NDVI = Normalised Difference Vegetation Index; PIWB = perennial inland water body.

**Figure 4:**
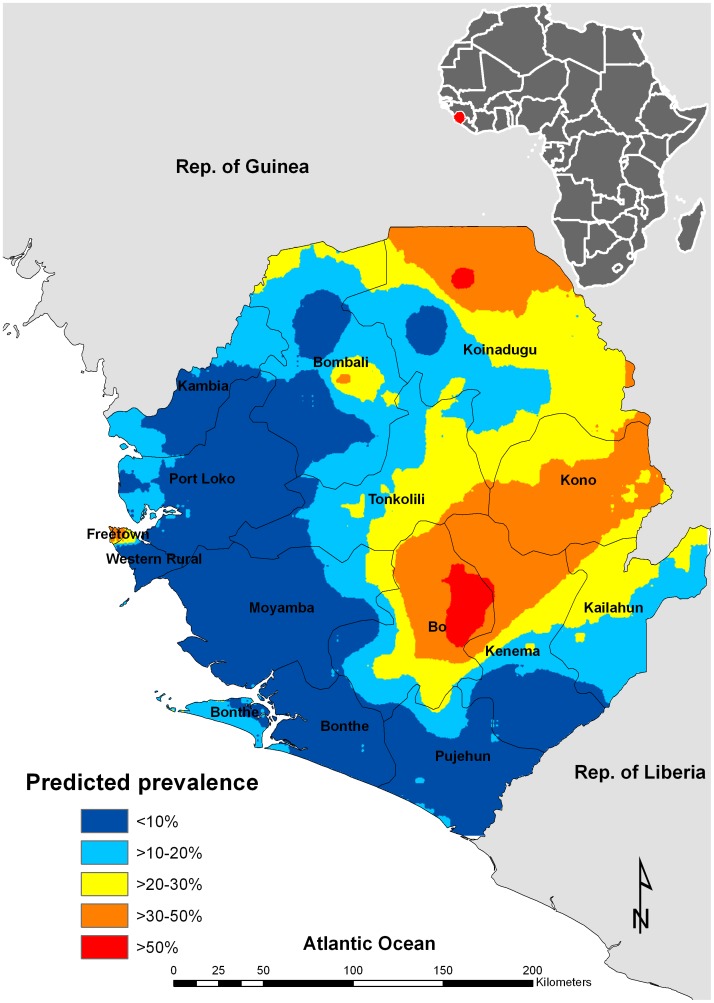
Predicted spatial distribution of urogenital schistosomiasis in Sierra Leone 2010.

The spatial prediction map showed a large area of moderate to high risk of *S. haematobium* infection (prevalence >10%) in the northeast two-thirds of the country, with clusters of significant risk of infection (prevalence >30%) in a region covering the northeast corner and an area spanning across Bo, Kenema and Kono districts of the country (Figure 4). The predicted prevalence was highest (prevalence >50%) in an area shared by the districts of Bo and Kenema and in a small cluster in the north of Koinadugu district. The model showed an acceptable predictive ability with an AUC of 0.78 (95%CI: 0.72–0.83).

### Combined Prevalence of Schistosomiasis

**Figure 5:**
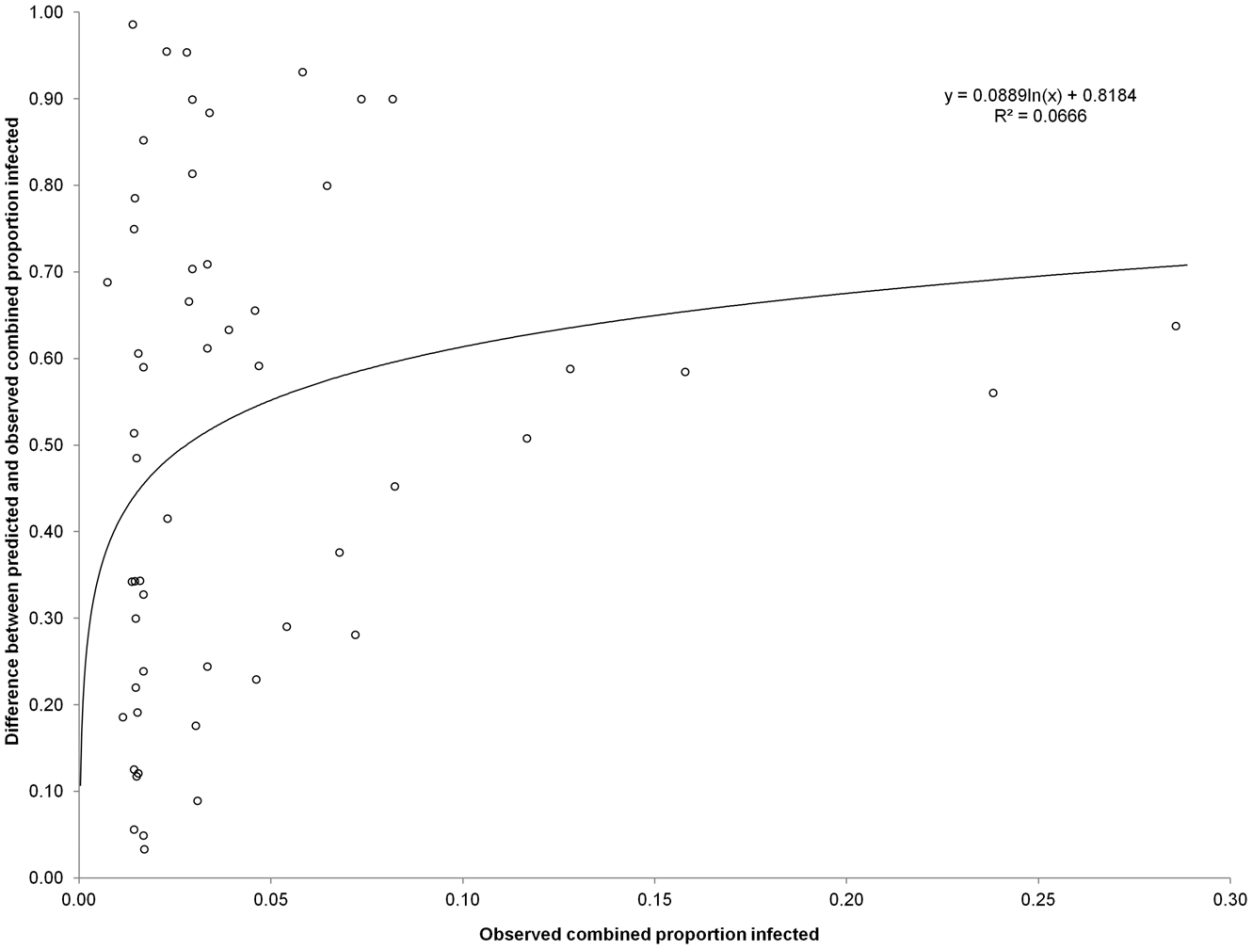
A scatter plot of the combined proportion with urogenital and intestinal schistosomiasis. The X-axis shows the observed combined proportion and the Y-axis shows the difference between the predicted and observed proportion infected.

The relationship between the difference between predicted and observed combined prevalence (*d_hm_*) against the observed combined prevalence (*o_hm_*) in each school was highly nonlinear (Figure 5). The best fitting function to the distribution was of the form:
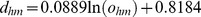
which indicates that the overestimation increased by 0.09 for every 10% increase in observed combined prevalence on the natural logarithmic scale.

**Figure 6:**
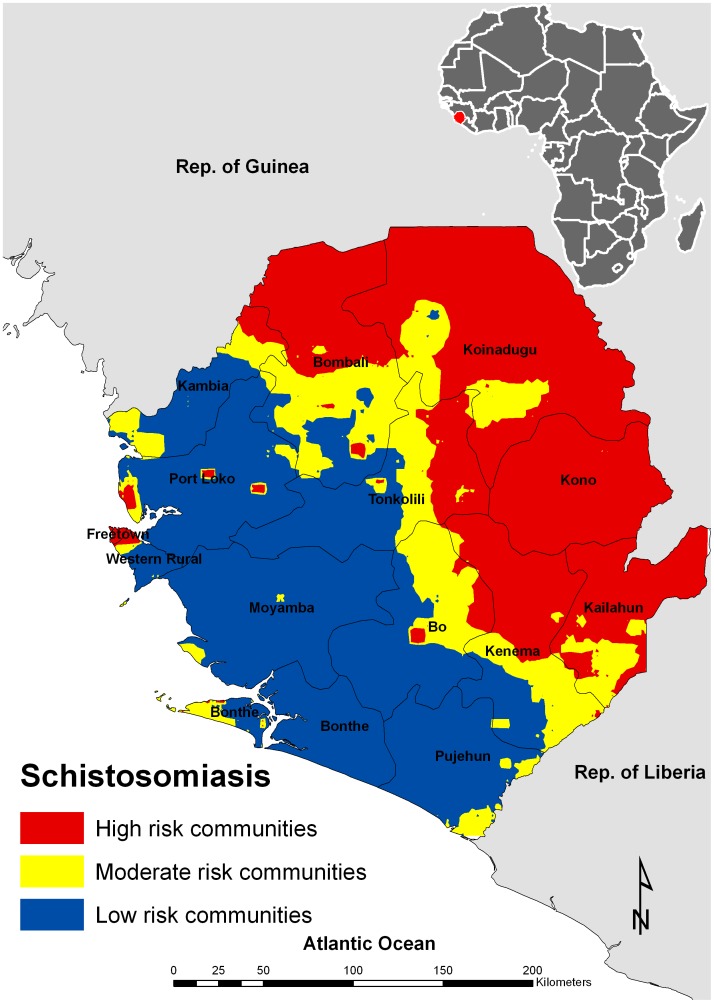
Spatial variation in combined schistosomiasis prevalence based on the WHO guidelines.

The combined schistosomiasis map for Sierra Leone highlights the presence of high risk communities in an extensive area in the northeastern half of the country (Figure 6). The transition between schistosomiasis risk areas is made in a northeast to southwest direction in the central districts of Bombali, Tonkolili, Bo and Kenema. Low risk areas (<10%) occupy most of the coastal and central districts of Sierra Leone.

### Number of School-Age Children with Schistosomiasis/Hookworm Infections

**Figure 7:**
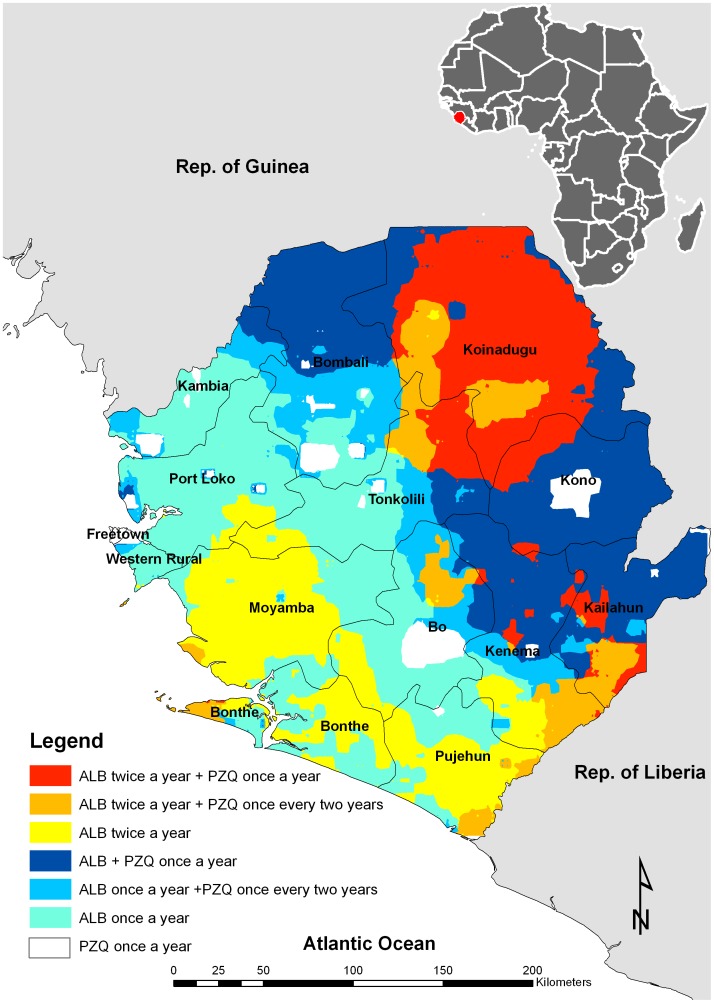
Spatial variation in treatment regimens for schistosomiasis and hookworm based on the WHO guidelines.

We predicted an extensive geographical overlap between the risk of schistosomiasis and hookworm. Based on the developed schistosomiasis/hookworm coendemicity map we constructed an integrated treatment map (Figure 7) which shows that most communities in the district of Koinadugu and smaller areas in the districts of Kailahun and Kenema will require once annually treatment of praziquantel and twice annually treatment of albendazole. In addition, school-age children in the districts of Moyamba, Bonthe, Pujehun, and few communities of southern Kenema will require twice a year treatment with albendazole. Most of the coastal and central areas of Sierra Leone will require albendazole once a year.

We estimated a total of 1,845,437 school-age children in Sierra Leone are in need of anthelminthic treatment of which 825,871 are in need of annual treatment of praziquantel; 56.5% (466,575) of those will also need albendazole once a year (Table S1). We also estimated that 302,814 school-age children will require praziquantel once every two years, and 61.3% (185,713) of those will also require a dose of albendazole once each year. Finally, we estimate that 716,752 school-age children in Sierra Leone will need praziquantel twice during their primary schooling age, and the majority of these (68.3%) will also need albendazole once each year.

## Discussion

This is the first comprehensive national mapping of urogenital schistosomiasis in Sierra Leone. The results were broadly in line with the previous data which showed that *S. haematobium* is heterogeneously distributed in the country with significant spatial clustering in the central and eastern regions of the country [8]. The current results confirmed that the population, particularly children, in these parts of the country is not only at risk of *S. mansoni* infection [9], [10] but also *S. haematobium* infection, further justifying the MDA for schistosomiasis in these seven endemic districts.

A collection of historical data showed that both urogenital and intestinal schistosomiasis were endemic in overlapping regions in Sierra Leone, but the former was more widely distributed with higher prevalence than the latter [7], [8]. There was an indication in the 1980s that *S. mansoni* was spreading in the country because of cross-border population movement and creation of snail habitats due to alluvial mining activities [7], [12]. The present results showed that, although both species are still endemic in the overlapping areas, *S. haematobium* has become a much less dominant species than *S. mansoni* [9], [10], and in fact it seems that *S. haematobium* may have been in decline. It is not clear why this shift in dominance has occurred in the last decades. Studies have shown that male *S. haematobium* worms are more dominant when competing with male *S. mansoni* worms when pairing with females in mixed infections [26], [27], but this contradicts the current findings. The switch may have been due to ecological reasons (e.g. snail habitats) rather than biological interactions between two species. Swamp rice farming was a major factor in the dramatic increase of schistosomiasis in the neighboring country Liberia [28], and such farming was encouraged in Sierra Leone but was found not to be spreading *S. mansoni* infection [7]. Mass human population movement in the rural districts may have led to the change in transmission dynamics and pattern causing the switch in species dominance.

Mapping of a disease distribution is a key step in planning an integrated national NTD control program. Schistosomiasis is a focal disease, with risk being closely related to the distance to the water sources where the intermediate host snails thrive [29]. Given the nature of the distribution, mapping of schistosomiasis has always attracted discussion in the current integrated control programs. Different strategies have been used in different countries: large scale surveys through as many schools as possible using Lot Quality Assurance Sampling method [30], [31], or stratified sampling surveys in selected schools using a geostatistical design and spatial interpolation [32], [33] have been proposed. Recent comparisons have showed that Lot Quality Assurance Sampling performs better than geostatistical sampling in correctly classifying schools, but at a higher cost per high prevalence school correctly classified [34]. It is always a balancing act between the program needs and the financial resources available when deciding the strategy for schistosomiasis mapping and how many sites to be surveyed. In Sierra Leone, original mapping surveys were designed based on the previous WHO recommendations [9], [19]. This proved to be insufficient for decision making at sub-district level, therefore, further surveys were conducted as described previously [10], and in this paper.

By combining all the data obtained throughout the country and overlaying the maps from different species, we were able to provide the most comprehensive understanding of distribution of schistosomiasis and hookworm in Sierra Leone and therefore optimal strategies for targeting of MDA. It is noted that the current coendemicity map for schistosomiasis was constructed using data from separate surveys for urogenital and intestinal schistosomiasis. Due to practical reasons, the surveys for the two species were conducted separately. To avoid overestimation or underestimation of the combined schistosomiasis prevalence by simple overlaying of different endemicity maps, we calculated the combined prevalence using a simple probabilistic model [23]. This model assumes independence between infections and this study indicates that in the case of schistosomiasis, this assumption would grossly overestimate the treatment needs by almost a million school-age children. To address this, the report has presented a new method for calculating the combined prevalence of schistosomiasis using estimates from two separate surveys which accounts for the highly non-linear relationship between observed and predicted combined prevalence of schistosomiasis and is therefore a more robust extension of the coendemicity mapping approach presented in an earlier study. Given the situation where the overall prevalence of schistosomiasis cannot be obtained for each community (which is typical for NTD control programmes in Sub-Saharan Africa), such coendemicity maps would provide a very useful tool to inform decisions for planning national MDA.

There are certain limitations in this study and the predicted coendemicity map. For the estimated number of school-age children, the population map used was based on the projected population. There may be a significant underestimate of the current population in Sierra Leone as the country underwent a significant population growth after the civil war and this was evident during MDA in the national NTD programme compared with the 2004 national census [35], [36]. Secondly, large migrations of internally displaced persons as a result of the civil war during 1991–2002 occurred initially from the east and then the north moving further towards the south and west. Many of these internally displaced persons have remained in the WA and other coastal districts post-war. The schistosomiasis cases identified in these surveys, particularly in the WA, may have been imported from more highly schistosomiasis-endemic districts. All children with *S. haematobium* infection in coastal districts in this survey were confirmed to be from internally displaced families (Hodges, personal observation). Indeed many of these internally displaced children are known to return to more highly prevalent districts during vacations to stay with their extended family there and then return to schooling in the low-prevalent coastal districts. Therefore, schistosomiasis endemicity in the WA and the coastal districts may have been overestimated, as there was no schistosomiasis or evidence of snails in these districts according to the historical data. Thirdly, the non-random selection of sites for *S. haematobium* surveys, which was based instead on historical data and local knowledge, may have led to overestimation of overall level of *S. haematobium* endemicity in the country. However, building on the previous *S. mansoni* and STH mapping [9], [10], and due to the specific nature of focal distribution of the disease, such purposeful and non-random sampling provided the national program with practical tools for MDA planning.

From the programmatic point of view, the current co-endemicity map should be used in conjunction with not only the local knowledge as described above, but also the overall programme needs when planning MDA in these districts. Implementation of MDA for schistosomaisis and STH in Sierra Leone is performed by different government Ministries (health and education), through different platforms (community-based and school-based), with different donors and different budget time-lines, functioning within different implementation units (chiefdoms versus districts), and overlapping with other NTD programs such as MDA with ivermectin and albendazole for lymphatic filariasis and/or onchocerciasis. In the context of integrated NTD control, planning of MDA for schistosomiasis and STH as indicated in the co-endemicity map needs to be coordinated to avoid repetition and to increase cost-efficiency.

In conclusion, the first comprehensive national mapping of urogenital schistosomiasis in Sierra Leone was conducted which showed that *S. haematobium* is heterogeneously distributed in the country with significant spatial clustering in the central and eastern regions of the country. Using a new method for calculating the combined prevalence of schistosomiasis using estimates from two separate surveys, we provided a robust coendemicity mapping for overall urogenital and intestinal schistosomiasis. We also produced a coendemicity map of schistosomiasis and hookworm. These coendemicity maps can be used to guide the decision making for MDA strategies in combination with the local knowledge and programme needs.

## Supporting Information

Text S1 **Statistical notation of Bayesian geostatistical models, spatial interpolation and model validation procedures for** ***Schistosoma haematobium*** **in Sierra Leone.**
(DOC)

Table S1 **Praziquantel and albendazole needs for integrated treatment of schistosomiasis and hookworm in Sierra Leone.**
(DOC)
