# Supplementary material for: Combined Spatial Prediction of Schistosomiasis and Soil-Transmitted Helminthiasis in Sierra Leone: A Tool for Integrated Disease Control
Source: PLoS Negl Trop Dis. 2012 Jun 19;6(6):e1694. doi: 10.1371/journal.pntd.0001694 (PMC3378624; doi:10.1371/journal.pntd.0001694)
Supplement: Text S1 — Statistical notation of Bayesian geostatistical models, spatial interpolation and model validation procedures for Schistosoma haematobium in Sierra Leone. (DOC) [file pntd.0001694.s001.doc]

**Supplementary technical information**

*General considerations*

The outputs of Bayesian models, including parameter estimates and spatial prediction at unsampled locations, are distributions termed “posterior distributions”. The posterior distributions fully represent uncertainties associated with the parameter estimates. We summarised the posterior distributions in terms of the posterior mean and 95% Bayesian credible interval (CrI), within which the true value occurs with a probability of 95%. All environmental variables were standardised by subtracting the mean and dividing by the standard deviation. The resulting regression coefficients for these variables represent the change in terms of standard deviations in prevalence of infection that result from a change of one standard deviation in these variables.

*Model specifications*

For the purpose of Bayesian geostatistical modelling the number of children positive with *S. haematobium* is considered a binomial outcome variable *Y_j_*. The models used assume a conditional Binomial model where the prevalence of infection *p_j_*, given the location *j* of the sample survey is given by:

$$\log it(p_{j})=\alpha+\sum_{k=1}^{c} \beta_{k}\times x_{j}+u_{j}$$

where *T_j_* is the total number of children tested in location *j, α* is the intercept, *x_j_* is a matrix of environmental covariates, *β* is a matrix of coefficients and *u_j_* is a geostatistical random effect defined by an isotropic powered exponential spatial correlation function:

,

where *d_ab_* are the distances between pairs of survey locations *a* and *b*, and is the rate of decline of spatial correlation per unit of distance. Non-informative priors were used for *α* (uniform prior with bounds - and ) and the coefficients (normal prior with mean = 0 and precision = 1 × 10^-4^). The precision of *u_j_* was given a non-informative gamma distribution. A uniform prior was given to (lower bound=0.25 and upper bound=20).

In all models, a burn-in of 5,000 iterations was allowed, followed by 10,000 iterations where values for the intercept, coefficients and predicted probability of infection at the prediction locations were stored. Diagnostic tests for convergence of the stored variables were undertaken, including visual examination of history and density plots; convergence was successfully achieved after 5,000 iterations.

The predictions of the prevalence of infection were made at the nodes of a 0.042 x 0.042 decimal degree grid (approximately 5 km^2^) by interpolating the geostatistical random effect and adding it to the sum of the products of the coefficients for the fixed effects and the values of the fixed effects at each prediction location. The interpolation of the random effect was done using the *spatial.unipred* kriging function in WinBUGS; the *spatial.unipred* command implements Bayesian kriging [1] where the values of predicted prevalence of infection at unsampled locations are estimated (interpolated) independently of neighbouring values, as opposed to joint prediction which is conditional on the values of neighbouring unsampled locations.

*Model validation*

The discriminatory ability of the model was assessed by dividing the original survey locations into four random subsets and sequentially withholding the data from one subset (the validation subset) while building the models with the remaining data (the training subset) and predicting prevalence of infection for the validation locations. Model calibration and discrimination was determined by using the area under the curve of the receiver operating characteristic (AUC) [2]. The predicted prevalence of *S. haematobium* infection at survey sites was compared to the corresponding observed prevalence, dichotomized at 50% (to assess model predictive performance) and the AUC was used for the comparison. When the predicted prevalence of *S. haematobium* infection at survey sites cannot distinguish between the two groups of observed prevalence, i.e. where there is no difference between the two distributions, the AUC will be equal to 0.5. When there is a perfect separation of the values of the two groups, i.e. there no overlapping of the distributions, the area under the AUC equals 1. An AUC value of 0.7 was taken to indicate acceptable predictive performance.

Supplementary tables

| Prevalence of urogenital schistosomiasis | Number of sites | Mean population numbers | Mean land surface temperature in ⁰C | Mean DPWB in km | Mean elevation |
| --- | --- | --- | --- | --- | --- |
| <20 | 39 | 4,724 | 9 | 75 | 131 |
| 20-50 | 12 | 1,632 | 17 | 65 | 250 |
| >50 | 1 | 1,125 | 18 | 199 | 577 |

*References*

1. Thomas A, Best N, Lunn D, Arnold R, Spiegelhalter D (2004) GeoBUGS User Manual version 1.2. Cambridge: Medical Research Council Biostatistics Unit.

2. Brooker S, Hay SI, Bundy DA (2002) Tools from ecology: useful for evaluating infection risk models? Trends Parasitol 18: 70-74.
