# Supplementary material for: Combined Spatial Prediction of Schistosomiasis and Soil-Transmitted Helminthiasis in Sierra Leone: A Tool for Integrated Disease Control
Source: PLoS Negl Trop Dis. 2012 Jun 19;6(6):e1694. doi: 10.1371/journal.pntd.0001694 (PMC3378624; doi:10.1371/journal.pntd.0001694)
Supplement: Table S1 — Praziquantel and albendazole needs for integrated treatment of schistosomiasis and hookworm in Sierra Leone. (DOC) [file pntd.0001694.s002.doc]

Table S1. Praziquantel and albendazole needs for integrated treatment of schistosomiasis and hookworm in Sierra Leone

| **Schistosomiasis prevalence threshold for MDA*** | **Hookworm prevalence threshold for MDA*** | **Target population and frequency** | **Number of children to be treated (%)†** | **Action to be taken to other members of the population** |
| --- | --- | --- | --- | --- |
| >50% | *All* | A. Treat all children with PZQ once a year | 825,871 | I. Also treat adults considered to be at risk (from special risk groups from entire communities) |
|  | >50% | A + Treat all children with ALB twice each year | 117,967  (14.3%) | I + Also treat: preschool children; Women of childbearing age; pregnant (2nd and 3rd trimesters); Lactating mothers; high risk adults – occupational risk adults (e.g. farmers, fresh water fishermen and miners) |
|  | >20-50% | A + Treat all children with ALB once each year | 466,575 (56.5%) | I + Also treat: preschool children; Women of childbearing age; pregnant (2nd and 3rd trimesters); Lactating women; high risk adults – occupational risk adults (e.g. farmers, fresh water fishermen and miners) |
| >10-50% | *All* | B. Treat all children with PZQ once every two years | 302,814 | II. Also treat adults considered to be at risk (from special risk groups only) |
|  | >50% | B + Treat all children with ALB twice each year | 62,247 (20.6%) | II + Also treat: preschool children; Women of childbearing age; pregnant (2^nd^ and 3^rd^ trimesters); Lactating women; high risk adults – occupational risk adults (e.g. farmers, fresh water fishermen and miners) |
|  | >20-50% | B + Treat all children with ALB once each year | 185,713 (61.3%) | II + Also treat: preschool children; Women of childbearing age; pregnant (2^nd^ and 3^rd^ trimesters); Lactating women; high risk adults – occupational risk adults (e.g. farmers, fresh water fishermen and miners) |
| <10% | *All* | C. Treat all children with PZQ (e.g. entry and exit of school) | 716,752 | III. Praziquantel should be available in dispensaries and clinics for the treatment of suspected cases. |
|  | >50% | C + Treat all children with ALB twice each year | 208,107 (29.0%) | III + Also treat: preschool children; Women of childbearing age; pregnant (2^nd^ and 3^rd^ trimesters); Lactating women; high risk adults – occupational risk adults (e.g. farmers, fresh water fishermen and miners) |
|  | >20-50% | C + Treat all children with ALB once each year | 489,206 (68.3%) | III + Also treat: preschool children; Women of childbearing age; pregnant (2^nd^ and 3^rd^ trimesters); Lactating women; high risk adults – occupational risk adults (e.g. farmers, fresh water fishermen and miners) |

*by parasitological methods

†Assumes a population growth rate 2005-2011 of 2.60% and proportion of 5-15 years old of 26.5% for 2011. Source: Population Division of the Department of Economic and Social Affairs of the United Nations Secretariat, *World Population Prospects: The 2010 Revision*, <http://esa.un.org/unpd/wpp/index.htm>

Abbreviations: MDA- mass drug administration; PZQ – praziquantel; ALB - albendazole
